# The Hybrid Reversible Suture‐Gripping Technique: A Modified Approach for Continuous Meniscal Suturing

**DOI:** 10.1002/atn2.70090

**Published:** 2026-06-01

**Authors:** Ryo Sasaki, Fuzan Maeda, Taichi Nishimura, Teppei Hayashi, Kazuya Kaneda, Masaki Nagashima, Hideo Morioka

**Affiliations:** ^1^ Department of Orthopaedic Surgery NHO Tokyo Medical Center Meguro‐ku Japan; ^2^ Department of Orthopaedic Surgery Keio University School of Medicine Shinjuku‐ku Japan; ^3^ Department of Orthopaedic Surgery International University of Health and Welfare Mita Hospital Minato‐ku Japan

## Abstract

The suture‐gripping technique (SGT), which uses a suture shuttle, enables unrestricted gripping, accurate needle insertion from inside the joint, and flexible withdrawal of the suture from outside the joint. We considered the possibility of using a continuous suturing technique with this method, and thus, we describe a modified approach, the hybrid reversible SGT, which allows bidirectional suture manipulation and facilitates continuous meniscal suturing. This technique uses the hybrid reversible SGT with locking stitches (baseball stitch) to repair the lateral meniscal tear. Each subsequent stitch is passed beneath the loop of the previous stitch to create a locked continuous suture with stable tension distribution along the repair line. Despite multiple passes, only 2 knots, 1 at each end, are required. This technique combines the flexibility and precision of the SGT with the biomechanical advantages of continuous suturing, providing secure fixation, minimal knot bulk, and efficient meniscal repair. Hybrid reversible SGT represents a practical and versatile evolution of a previously reported technique, extending its applicability to complex meniscal tears.

## Graphical abstract

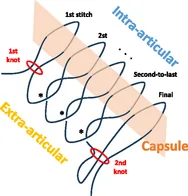

**VIDEO 1:** The suture‐gripping technique (SGT) allows unrestricted gripping and flexible withdrawal of sutures from outside the joint, providing accurate needle control and improved maneuverability. Building on this concept, we describe a modified approach, the hybrid reversible SGT (HR‐SGT), which enables bidirectional suture manipulation and facilitates continuous meniscal suturing. First, the SGT is shown. The loop of the suture shuttle is expanded, and the suture is passed through the loop. The loop is then closed, and the suture is gripped with the needle tip. The case is a 20‐year‐old male who injured his right knee 1 month prior. Under general anesthesia, diagnostic arthroscopy is first performed through the standard anterolateral portal. A longitudinal tear of the discoid lateral meniscus is identified. After saucerization, a longitudinal tear extending from the anterior to the posterior portions is visualized; therefore, the posterior portion is repaired using the all‐inside technique, with the knee in the figure‐4 position. Next, the longitudinal tears of the anterior and middle segments are repaired using the HR‐SGT continuous locking method. As the standard anteromedial portal does not provide an adequate trajectory for the suture shuttle toward the anterior horn, a far‐medial portal is created. A 2‐cm skin incision is made at the planned suture site, and the subcutaneous tissue is carefully dissected to expose and identify the joint capsule. Continuous suturing with the HR‐SGT begins from the lateral direction toward the medial direction. Under arthroscopic visualization through the anteromedial portal, the suture shuttle is passed from the proximal capsule near the meniscus to the extra‐articular space. The loop of the shuttle is then expanded, and the suture is passed through it. The loop is closed, and the suture is gripped with the needle tip. With the suture held in this position, the suture shuttle is advanced back into the joint and then passed from the distal capsule near the meniscus to the extra‐articular space. The loop is expanded again, and the suture is released from the loop. The 2 suture limbs exiting the capsule are securely tied. The HR‐SGT is performed for the second stitch. During suture withdrawal into the extra‐articular space, the free end of the suture is passed beneath the loop of the first stitch and pulled, creating a locking configuration similar to that of a baseball stitch. This pattern ensures stable tension transmission and uniform compression along the repair line, maintaining alignment without additional knots. This procedure is repeated sequentially along the anterior segment of the lateral meniscus. Each new pass locks beneath the previous loop, creating a continuous locking, baseball‐style suture pattern that maintains consistent tension and alignment throughout the repair. After completion of the final stitch (5 stitches in this case), the final suture end is temporarily tied to the loop of the preceding stitch outside the capsule. Adequate tension across all stitches is confirmed arthroscopically, and final knot tying is then performed. Only 2 knots are required regardless of the number of stitches. Finally, the repaired meniscus is inspected arthroscopically to confirm anatomical reduction and stable fixation under appropriate tension. After confirming satisfactory stability, all portals are closed in a standard manner. Video content can be viewed at https://doi.org/10.1002/atn2.70090.

Meniscal preservation is essential for maintaining knee function and preventing degenerative joint changes.^1^ Various arthroscopic techniques have been developed for meniscal repair, including all‐inside, outside‐in, and inside‐out approaches.^1^

The suture‐gripping technique (SGT), which uses a suture shuttle, enables unrestricted gripping, accurate needle insertion from inside the joint, and flexible withdrawal of the suture from outside the joint, providing greater accuracy and maneuverability than conventional methods.^2^ Building on this concept, we describe a modified approach, the hybrid reversible SGT (HR‐SGT), which allows bidirectional suture manipulation and facilitates continuous meniscal suturing. This report describes the technical steps, advantages, and biomechanical rationale for this method (Video 1).

## SURGICAL TECHNIQUE

### Hybrid Reversible SGT

**FIGURE 1:**
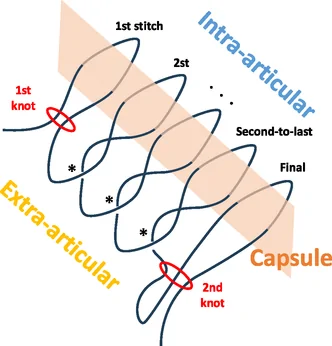
Step‐by‐step illustration of the HR‐SGT with locking stitches. The suture shuttle is first passed from inside the joint to the extra‐articular space at the desired repair site. With the suture held using the SGT (Figure 2), the shuttle is then advanced back into the joint and withdrawn again to the outside at the next repair point. The suture is released, and the 2 suture limbs exiting the capsule are securely tied. Subsequently, the shuttle is reintroduced into the joint. At the next repair site, it is withdrawn to the outside, where one of the previously tied sutures is gripped using the SGT and guided from the outside to the inside and then back to the outside of the joint. In this case, during withdrawal into the extra‐articular space, the free end of the suture is passed beneath the loop of the first stitch and pulled, creating a locking configuration similar to that of a baseball stitch. Continuous suturing is achieved by sequentially repeating this process. After completion of the final stitch, the suture exiting the joint is tied to the loop of the preceding stitch outside the capsule, completing the continuous suture with only 2 knots. (The black asterisks indicate the baseball stitch‐like locking configurations.) (HR‐SGT, hybrid reversible suture‐gripping technique.)

The HR‐SGT is a modified arthroscopic suturing method designed to enable continuous meniscal repair using bidirectional suture manipulation (Figure 1). This technique combines conventional SGT with a reversible shuttle maneuver, allowing the surgeon to guide the suture freely from the inside to the outside and back inside the joint, without repeated instrument exchanges.

**FIGURE 2:**
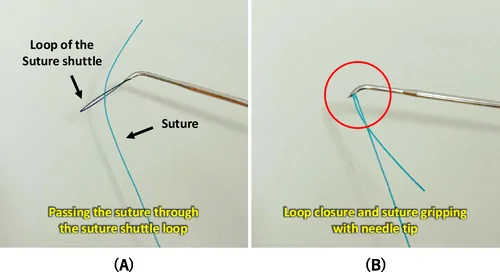
Suture‐gripping technique. (A) The loop of the suture shuttle is expanded, and the suture is passed through the loop. (B) Next, the loop is closed, and the suture is gripped with the needle tip.

First, a skin incision is made at the planned suture site. The subcutaneous tissue is carefully dissected to expose and identify the joint capsule. The suture shuttle is passed from the inside of the joint to the extra‐articular space at the desired repair site. The loop of the suture shuttle is then expanded, and the suture is passed through the loop. Next, the loop is closed, and the suture is gripped with the needle tip (i.e., the SGT) (Figure 2). With the suture held in this position, the suture shuttle is advanced back into the joint and then withdrawn again to the outside at the next desired repair point. The loop of the shuttle is expanded again, and the suture is released from the loop. The 2 suture limbs exiting the capsule are securely tied.

Subsequently, the suture shuttle is closed and advanced into the joint. At the next repair site, the suture shuttle is withdrawn to the outside, and one of the previously tied sutures is gripped using the SGT and guided from the outside to the inside and then back to the outside of the joint.

This maneuver allows the surgeon to freely guide the suture from the outside to the inside and then back outside the joint, enabling bidirectional suture manipulation. Continuous suturing is achieved by sequentially repeating the same process.

After completion of the final stitch, the suture exiting the joint is tied to the loop of the preceding stitch outside the capsule, completing the continuous suture with only 2 knots.

### Patient and Preparation

A patient presents a longitudinal tear in the lateral meniscus (LM). For preoperative preparation, magnetic resonance imaging was performed to evaluate the meniscal tears.

Under general anesthesia, the patient is placed in the supine position on an operating table with a standard leg holder, allowing a full range of motion. Complete diagnostic arthroscopy is initially performed through the anterolateral and anteromedial portals.

**FIGURE 3:**
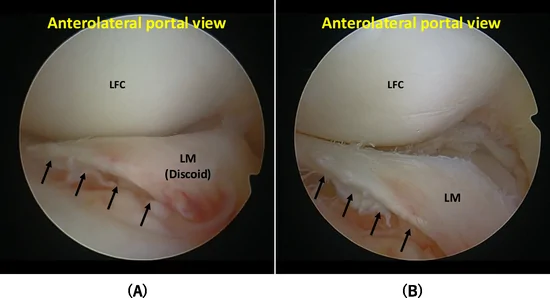
Diagnosis of meniscus injury and saucerization (anterolateral portal view; right knee in the figure‐4 position). (A) Longitudinal tear of the discoid LM (black arrows). (B) After saucerization of the discoid LM, a longitudinal tear extending from the anterior to the posterior body is identified (black arrows). Thus, the posterior segment of the LM tear is repaired with an all‐inside technique, and the anterior to middle segment is sutured using the hybrid reversible suture‐gripping technique. (LFC, lateral femoral condyle; LM, lateral meniscus.)

**FIGURE 4:**
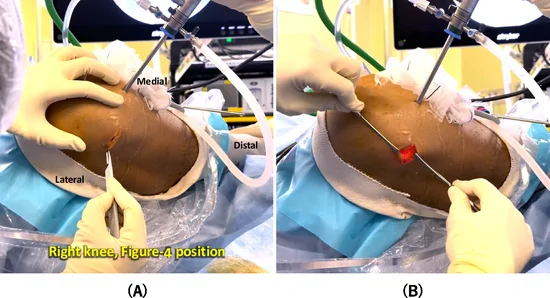
Skin incision and exposure of the capsule (lateral view of the right knee; figure‐4 position). (A) A 2‐cm skin incision is made at the planned suture site. (B) The subcutaneous tissue is carefully dissected to expose and identify the joint capsule.

A longitudinal tear of the discoid LM is observed (Figure 3A). After saucerization of the discoid LM, a longitudinal tear extending from the anterior to the posterior body is identified (Figure 3B); therefore, the posterior body of the meniscus and the posterior joint capsule are sutured using the all‐inside technique (FAST‐FIX FLEX; Smith & Nephew Endoscopy, Andover, MA, USA), with the knee in the figure‐4 position. Next, the longitudinal tears of the anterior and middle segments of the LM are sutured by continuous locking suturing using HR‐SGT with a suture shuttle (ACCU‐PASS Suture shuttle, 70° upbend; Smith & Nephew Endoscopy, Andover, MA, USA).

### Creation of a Far‐Medial Portal

Because the standard anteromedial portal does not allow an adequate trajectory toward the anterior horn, a far‐medial portal is created to facilitate an optimal shuttle angle for HR‐SGT suturing.

### Skin Incision and Exposure of the Capsule

A 2‐cm skin incision is made at the planned suture site (Figure 4A). The subcutaneous tissue is carefully dissected to expose and identify the joint capsule (Figure 4B).

### First Stitch: Initiation of the HR‐SGT

**FIGURE 5:**
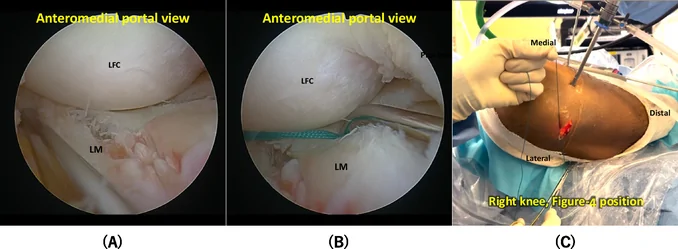
First stitch (initiation of the HR‐SGT) (anteromedial portal and lateral views of the right knee; figure‐4 position). Continuous suturing is initiated from the lateral toward the medial direction using the HR‐SGT. The proximal capsule near the meniscus (A) and the distal capsule adjacent to the tibial plateau (B) are sutured with a no. 2 Ethibond suture using a suture shuttle from the far‐medial portal, and the suture is tied outside the joint to secure the initial fixation (C). This step establishes the starting point for the continuous reversible sequence. (HR‐SGT, hybrid reversible suture‐gripping technique; LFC, lateral femoral condyle; LM, lateral meniscus.)

Continuous suturing is initiated from the lateral direction toward the medial direction using the HR‐SGT. Under arthroscopic visualization through the standard anteromedial portal, the proximal capsule near the meniscus and the distal capsule adjacent to the tibial plateau are sutured with a no. 2 Ethibond suture using a suture shuttle from the far‐medial portal (Figure 5A,B). Then, the suture is tied outside the joint to secure the initial fixation (Figure 5C), thus establishing the starting point for a continuous reversible sequence.

### Second Stitch and Locking Configuration

**FIGURE 6:**
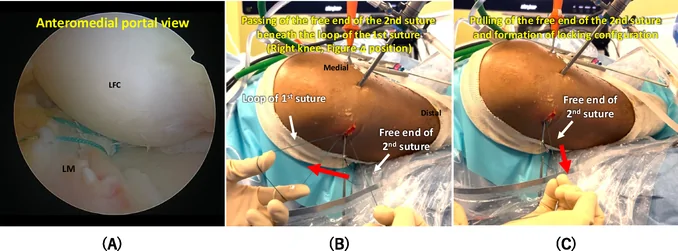
Second stitch and locking configuration (anteromedial portal and lateral views of the right knee, figure‐4 position). (A) The HR‐SGT is applied for the second stitch. During withdrawal of the suture to the extra‐articular space, the free end of the second suture is passed beneath the loop of the first stitch (B) and pulled (C), thereby creating a locking configuration (baseball stitch). This ensures stable tension transmission and uniform compression along the repair line. This locking pattern represents the continuous feature of the HR‐SGT, maintaining alignment without the need for additional knots. (HR‐SGT, hybrid reversible suture‐gripping technique; LFC, lateral femoral condyle; LM, lateral meniscus.)

The same technique is used for the second stitch. During suture withdrawal into the extra‐articular space, the suture is passed beneath the loop of the first stitch, thereby creating a locking configuration (baseball stitch) (Figure 6). This ensures stable tension transmission and uniform compression along the repair line. This locking pattern represents the “continuous” feature of the HR‐SGT, maintaining alignment without additional knots.

### Continuous Suturing

This procedure is sequentially repeated to achieve continuous suturing along the anterior segment of the LM. Each new pass locks beneath the previous loop, producing a continuous locking (baseball stitch) suture pattern that maintains consistent tension and alignment throughout the repair.

### Final Knot Tying and Arthroscopic Confirmation

**FIGURE 7:**
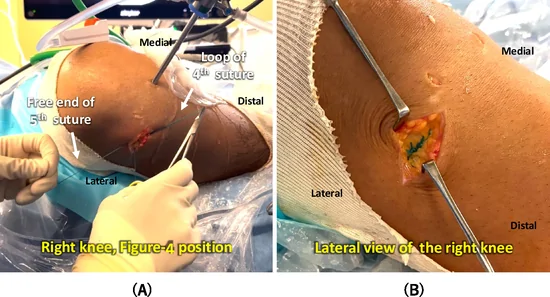
Final knot tying (lateral views of the right knee; figure‐4 and extended positions). (A) After completion of the final stitch (5 stitches in this case), the final suture end is temporarily tied to the loop of the preceding stitch (the fourth stitch) outside the capsule. (B) After arthroscopic confirmation of adequate tension across all stitches, the final knot is tied.

After completion of the final stitch (5 stitches in this case), the final suture end is temporarily tied to the loop of the preceding stitch (the fourth stitch) outside the capsule (Figure 7A). Arthroscopic visualization is used to confirm adequate tension across all stitches before final knot tying. Only 2 knots, 1 at the beginning and 1 at the end, are required, regardless of the number of stitches, resulting in a smooth, low‐profile construct (Figure 7B).

**FIGURE 8:**
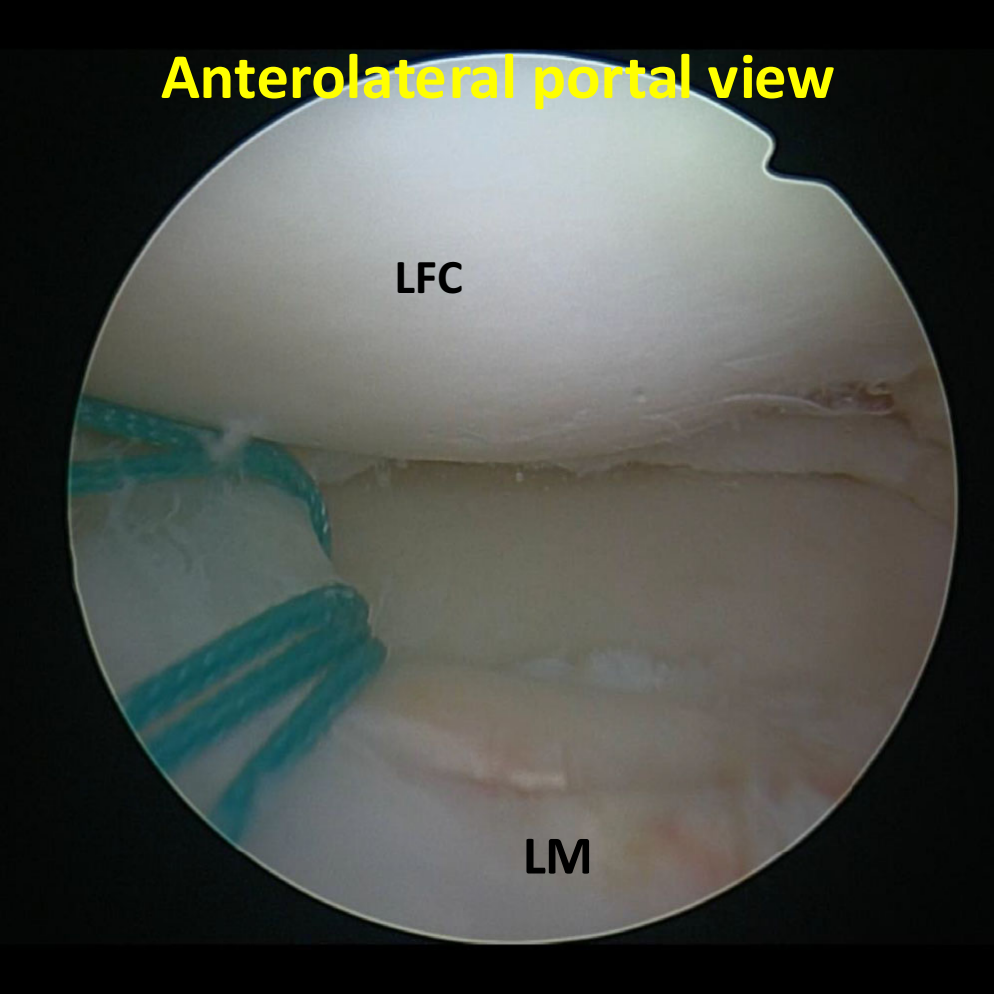
Final arthroscopic confirmation (anterolateral portal view; right knee in the figure‐4 position). The repaired meniscus is inspected arthroscopically to confirm anatomical reduction and stable fixation under appropriate tension. After confirming satisfactory stability, all portals are closed in the standard manner. (LFC, lateral femoral condyle; LM, lateral meniscus.)

The repaired meniscus is inspected arthroscopically under appropriate tension to confirm anatomical reduction and stable fixation (Figure 8). After confirming satisfactory stability, all the portals are closed in a standard manner (Table 1).

**TABLE 1:** Pearls and Pitfalls

Pearls	Pitfalls
Creates a far‐medial portal for better access to the anterior horn	Incorrect portal may cause poor needle trajectory
Make a 2‐cm incision to expose the capsule	Uneven tension can lead to gap or overconstraint
Pass each stitch beneath the previous loop for locking configuration	
Adjust tension arthroscopically before final knot tying	

## DISCUSSION

With advancements in arthroscopic equipment, various meniscal repair techniques have been developed.^1^ However, in the anterior segments of the meniscus, particularly when using the outside‐in technique for unstable tears, complex intra‐articular maneuvers and advanced surgical skills are often required, and there are restrictions on the type and diameter of the suture material that can be used.^3^, ^4^, ^5^, ^6^ Although the all‐inside technique is technically simple, it leaves knots within the joint cavity, which may cause articular cartilage irritation.^7^

To overcome these limitations, the SGT using a suture shuttle is performed, as it allows accurate needle insertion into the optimal meniscal position from inside the joint while flexibly guiding the suture from outside the capsule.^2^ Therefore, we describe a modified method, the HR‐SGT, which enables bidirectional continuous suture manipulation, allowing the surgeon to guide the suture from outside to inside and then back to the outside of the joint.

The HR‐SGT combines the technical flexibility of the SGT with the mechanical advantages of continuous suturing (Table 2). The most notable benefit is that although multiple stitches are placed, only 2 knots—1 at the beginning and 1 at the end—are required. This feature minimizes extra‐articular knot bulk and reduces the risk of soft‐tissue irritation. Furthermore, by passing each subsequent stitch beneath the loop of the previous stitch, a locked continuous configuration is achieved, ensuring stable tension transmission and uniform compression along the repair line.

**TABLE 2:** Advantages and Disadvantages

Advantages	Disadvantages
Allows continuous (baseball‐style) suturing with stable tension	Limited access to meniscal areas unreachable by the suture shuttle
Requires only 2 knots, reducing knot bulk and irritation	Difficult in obese patients owing to limited suture shuttle reach
Provides accurate and flexible needle control from inside the joint	
Permits use of any suture material without diameter restriction	
Enables easy access to anterior horn using a compact device	
Creates extra‐articular knots, preventing cartilage damage	

Previous reports have described various methods of continuous meniscal suturing.^8^, ^9^, ^10^, ^11^, ^12^, ^13^ Although these methods achieve secure fixation, they generally require complex maneuvers and ultimately require extra‐articular knot‐tying for each stitch. In contrast, the HR‐SGT allows continuous locking suturing with only 2 terminal knots, thereby simplifying the procedure while maintaining strong fixation.

Another major advantage of the HR‐SGT is its technical versatility and material flexibility. Because the suture shuttle can freely grip and retrieve sutures of any material, there are no restrictions on suture type or thickness. In addition, the compact design and controllability of the suture shuttle facilitate access to the anterior horn of the meniscus, which is difficult to reach with conventional devices. If the meniscus cannot be accessed via standard anteromedial or anterolateral portals, creating a far‐medial or far‐lateral portal allows for optimal needle trajectory and suturing accuracy.

Biomechanically, continuous locking sutures distribute tension evenly across the repair line, reducing localized stress and minimizing the risk of gap formation between stitches. External placement of knots eliminates intra‐articular knot impingement, thereby preventing articular cartilage irritation and improving postoperative outcomes. Therefore, this technique provides both mechanical stability and biological safety, which are the key factors for successful meniscal healing. Although uniform tension distribution is advantageous for stable fixation, slight tension adjustment can be achieved under arthroscopic visualization before final knot tying. This allows the surgeon to optimize suture tightness according to local meniscal tissue condition and tear configuration.

Although the final construct may appear relatively loose in static arthroscopic images, appropriate tension is confirmed intraoperatively by probing and dynamic assessment under arthroscopic visualization. Importantly, excessive tightening should be avoided in techniques that involve fixation of the meniscus to the joint capsule, as overtensioning may lead to meniscal extrusion and alter normal knee biomechanics.

With regard to the use of only 2 knots, the locking configuration created by the continuous baseball stitch‐like technique provides load sharing across the repaired segment. In addition, the surgeon may add supplementary half‐hitches or an additional knot at any repair site if insufficient stability is observed intraoperatively.

Nonetheless, this study has a limitation that must be acknowledged. This method may not be applicable to meniscal regions that are anatomically unreachable by suture shuttle, even when additional portals are created. In addition, this report is based on a limited number of clinical cases; therefore, further studies with larger cohorts and longer follow‐up periods are needed to evaluate the long‐term clinical outcomes and reproducibility of the HR‐SGT. Despite this limitation, HR‐SGT is a highly useful and versatile approach that offers enhanced flexibility, minimal knot burden, and a strong, uniform fixation suitable for complex anterior meniscal tears.

In conclusion, the HR‐SGT combines the advantages of the inside‐out and outside‐in approaches with the biomechanical benefits of continuous locking suturing, providing a simple, reproducible, and efficient method for meniscal repair.

## DISCLOSURES

The authors (R.S., F.M., T.N., T.H., K.K., M.N., H.M.) declare that they have no known competing financial interests or personal relationships that could have appeared to influence the work reported in this article.
